# Metabarcoding identifies macroalgal composition as a driver of benthic invertebrate assemblages in restored habitats

**DOI:** 10.1038/s41598-025-93327-4

**Published:** 2025-03-21

**Authors:** Cristina Galobart, Jesús Zarcero, Adrià Antich, Xavier Turon, Emma Cebrian

**Affiliations:** 1 Department of Marine Ecology, Centre d’Estudis Avançats de Blanes (CEAB-CSIC), Accés Cala Sant Francesc 14, Blanes, (17300) Spain; 2 NORCE Norwegian Research Centre AS and Bjerknes Centre for Climate Research, 22 Nygårdstangen, Bergen, (NO-5838) Norway

**Keywords:** Restoration success indicators, Macroalgal restoration, Algal-invertebrate interactions, Metabarcoding, Cytochrome oxidase I, *Gongolaria barbata*, Marine biology, Molecular ecology

## Abstract

Large brown macroalgal forests support diverse communities of associated invertebrates. However, human activities have led to their degradation, replacing the original macroalgal assemblages with less-complex habitats and altering the associated invertebrate communities. To counteract this, restoration actions are currently being performed aiming at their recovery. Here, we used metabarcoding of the COI gene to describe the invertebrate community of a 10-year restored macroalgal forest dominated by *Gongolaria barbata*. This invertebrate assemblage was compared with an assemblage adjacent to the restoration site where *G. barbata* is absent, and two healthy assemblages also dominated by *G. barbata*, used as references. Results showed that arthropods, molluscs, and annelids were the most abundant groups. Specific MOTUs representing sponge and ascidian species were exclusively found in *G. barbata* assemblages, suggesting that the restoration particularly benefited some of these organisms. Crustaceans and molluscs, however, were similarly abundant across assemblages, including the one dominated by photophilous macroalgae (without *G. barbata*), thus indicating that these mobile groups may equally colonise these alternative habitats. The overall composition of the restored assemblage did not completely resemble references, suggesting that recovering all associated diversity is a complex process, most likely influenced by multiple factors that require further investigation.

**Supplementary Information:**

The online version contains supplementary material available at 10.1038/s41598-025-93327-4.

## Introduction

Macroalgal canopy-forming species belonging to the order Laminariales, Fucales, and Tilopteridales dominate the low intertidal and subtidal rocky bottoms, forming extensive seaweed habitats known as marine forests^1^. They play a crucial role in marine ecosystems by modifying their physical environment, increasing three-dimensional structure and providing food and shelter for many other organisms^2,3^. Moreover, they provide a wide variety of ecosystem services to humans, such as reduction in coastal eutrophication and support to commercial fisheries^4–6^. In the Mediterranean Sea, these forests are typically characterised by species of the genera *Cystoseira sensu lato* (including *Cystoseira* C. Agard, *Gongolaria* Boehmer and *Ericaria* Stackhouse)^7,8^, resulting in highly diverse and complex habitats^9,10^.

During the last decades, these marine forests have declined in many areas across the Mediterranean, which stands out among the most impacted marine regions globally^11,12^. *Cystoseira* *s.l.* species are particularly vulnerable to human-induced disturbances, including coastal urbanisation and habitat destruction, as well as overgrazing by sea urchins and herbivorous fish^13,14^. The cumulative effects of these stressors have led to the local extinction or significant reduction of once widespread populations^15^. As a result, the original assemblages of macroalgal species within *Cystoseira s.l.* forests are often substituted by alternative habitats such as turfs or barren grounds, which are simpler and less productive communities^16,17^. In other cases, *Cystoseira s.l.* forests are replaced by other photophilous macroalgal species, such as erect macroalgae and shrub species, which represent an intermediate level of structural complexity^18,19^. Previous studies have observed that macroalgal identity may determine the associated algal and macroinvertebrate species that colonise these environments^20–22^. However, the full extent to which these shifts in macroalgal species influence the dynamics of the entire ecosystem in terms of associated biodiversity, is still not completely understood.

On the other hand, as an effort to mitigate the decline of marine forests, management strategies have increasingly considered initiatives of ecological restoration^23–25^, particularly adequate when natural recovery of these populations is hindered even after removing the causes that led to the decline in the first place (e.g^16^). Several protocols and methodologies are now being developed in order to improve and scale up restoration actions (e.g^26,27^). However, most current examples of macroalgal restoration are usually monitored for less than 2 years^23,24^, potentially resulting in masked or misinterpreted findings^25^. Moreover, success evaluations are mainly focused on the growth and survival of the target species^28^. These factors, combined with other stochastic events such as coastal storms (e.g^29^), contribute to the overall unpredictability of macroalgal restoration success, a pattern commonly observed in global marine restoration, where outcomes also exhibit great variability^30,31^. Therefore, there is a strong need for practical examples to help understanding in which cases and to which extent restoration can reverse the degradation of marine forests. In this sense however, few studies have used the recovery of the associated biodiversity as a success indicator after restoration (e.g. macroalgae^32^; macroinvertebrates^33–35^). In particular, assessing the entire invertebrate species composition, encompassing all phyla within a community and at a species-level, can often be difficult to attain. Morphological identification of invertebrate fauna taxa is extremely challenging, owing to a combination of: (i) the broad diversity of many groups, (ii) the required taxonomic expertise, (iii) the presence of highly cryptic taxonomic groups (e.g. isopods, micro-gastropods), and (iv) the time needed for the identification of many samples^36–38^. Therefore, attempts to monitor the invertebrate communities associated with the change of the macroalgal composition are both highly time- and resource-consuming.

Alternatively, DNA metabarcoding is by now a consolidated approach for the assessment of biodiversity in marine sample^39^. This technique enables the simultaneous identification of many taxa within a sample based on high-throughput sequencing of genomic DNA amplicons^40^ obtained from different sources (water, sediment, bulk samples). Metabarcoding has revolutionised biodiversity studies in the marine environment in the last decade and is now widely applied in various fields such as biomonitoring^41^, invasion biology^42^ or food webs and trophic interactions^43^. It is acknowledged that the technique is subjected to several limitations, including the need for more comprehensive reference databases or the improvement and standardisation of methods and protocols, specifically for Eukarya^44,45^. Despite these challenges, DNA metabarcoding is currently an effective technique for quickly and consistently producing extensive biodiversity data. When identical protocols and markers are applied, the results are highly reliable and comparable.

In this study we aim to understand the patterns of invertebrate fauna biodiversity associated with different macroalgal assemblages, representing pre- and after-restoration states and reference targeted communities. To do so, we took advantage of a ten-year restoration action in Menorca (Balearic Islands, NW Mediterranean), and analysed the influence of macroalgal composition on the invertebrate communities. Applying metabarcoding of the Cytochrome Oxidase subunit I (COI) fragment and with a set of generalist primers targeting the eukaryotic communities, we compared four assemblages: the restored macroalgal forest (where the structural species *G. barbata* was recovered and it is now abundant), an assemblage similar to the one that was present before the restoration (mostly dominated by shrub photophilous algae), and two assemblages from healthy and mature *G. barbata* macroalgal forests.

## Methods

### Studied assemblages and sample processing

An example of a successful marine forest restoration is the one dealing with the structural species *Gongolaria barbata* (Stackhouse) Kuntze (Phaeophyceae), in the Menorca island (NW Mediterranean)^46^. In this island, *G. barbata* used to be abundant in the two main, large bays: the Bay of Fornells and the Bay of Maó. During the 1970s, as a consequence of a decline in water quality due to direct release of urban sewage and harbour activities in the area, *G. barbata* disappeared from the Bay of Maó. In 1980, a sewage plan was built and, although it led to a significant improvement in water conditions, no recovery of the species was detected^47^. In 2011, an active restoration action targeting the structural species was performed in the Bay of Maó (see^46^ for a complete description of the process). This action successfully restored the density and size structure distribution of *G. barbata*^46^ along with the associated macroalgal species^32^, and resulted in a restored area of ca. 2,000 m^248^.

The Bay of Fornells and the Bay of Maó are highly sheltered bays with minimal freshwater input and a substrate consisting of both sand and rocks. These bays and the habitats they support match the description of the habitat classification “*Large shallow inlets and bays*,* code 1160*” of the European Habitats Directive (Council Directive 92/43/EEC). In the Mediterranean, these particular environmental characteristics allow for the coexistence of macroalgal and seagrass species in the upper infralittoral zone^49,50^. Specifically, in the Bay of Fornells and the Bay of Maó, several *Cystoseira s.l.* species grow together with the seagrass *Cymodocea nodosa*. The infralittoral zone is highly sheltered and shallow (less than 1 m depth), with gentle slope and abundant irradiance, supporting habitats classified in the Balearic Islands as “infralittoral with *Gongolaria barbata/Cystoseira foeniculacea* var. *tenuiramosa*” (code 0301030601;^51^).

In this study, we compared four different assemblages within the two large Bays of Menorca island (Supplementary Figure S1). In the Bay of Maó, we examined the restored assemblage (referred to as “Restored Forest”), which represents the *G. barbata* community 10 years after the restoration action. We also studied an assemblage located near the restoration action (~ 50 m apart), where *G. barbata* is still absent and corresponds to the community present before the restoration action (referred to as “Not Restored”). Healthy and mature *G. barbata* assemblages were sampled in the bay of Fornells, one in Cala Rotja and another in Miami (referred to as “Cala Rotja Forest” and “Miami Forest”, respectively), and were included in this study for comparison purposes with the Restored Forest assemblage.

Three replicates of 25 × 25 cm per assemblage were scraped to bare rock with hammer and chisel and collected in plastic bags. Samples were immediately fixed on site by eliminating water through a sieve of 63 μm mesh size and replacing it with absolute alcohol. Samples were stored at -20 ºC until processing. Before processing in the laboratory, we carefully measured the biomass of the most abundant macrophyte species in each sample, measured as wet weight. The species were selected according to the results obtained in a previous study dealing with the same assemblages, collected at the same time of the year^32^. The weighted species were: *Cystoseira s.l.* species, *Cymodocea nodosa*, *Padina pavonica*, and *Halopteris scoparia*. After this, each scrapped sample was separated under gentle freshwater flow into two different fractions (hereafter “Large” and “Small”) using a column of two stainless-steel sieves of 1 mm and 63 μm mesh size, respectively^52^. Each fraction was then placed in absolute ethanol and homogenised with a blender. Sample fractionation is necessary to ensure a correct representation of small organisms. Given the big differences in size of the collected organisms, the DNA pool would otherwise be dominated by large ones, hindering the detection of those from smaller size classes. As big organisms are retained in the “Large” fraction, DNA copies of smaller organisms are more concentrated in the “Small” fraction, thereby enhancing their detection^52,53^. Three negative controls were prepared by charring sand samples in a muffle furnace at 450ºC for 4 h, followed by sieving and processing them in the same manner as the other samples. All equipment was carefully bleached between samples. Our final sample dataset consisted of 24 samples (four assemblages x three replicates x two fractions).

### DNA extraction, amplification and sequencing

The procedure to obtain DNA sequences followed^54^. For DNA extraction, 5 g of each homogenate was processed with the DNeasy PowerMax Soil Kit (Qiagen). We amplified a ca. 313 bp fragment of the Cytochrome Oxidase C subunit 1 (COI) gene using generalist primers designed for eukaryotes. In particular, we used the Leray-XT primer set^52,55^ which consists of the forward primer jgHCO2198: 5’-TAIACYTCIGGRTGICCRAARAAYCA-3’ and the reverse primer mlCOIintF-XT: 5’-GGWACWRGWTGRACWITITAYCCYCC-3’^52^. An 8-base tag was added at the 5’ end of the primers. Unique tags were assigned to each sample, ensuring a minimum difference of 3 bases between tags, with the same tag applied to both forward and reverse primers to simplify the removal of inter-sample chimeras. Degenerate bases (from 2 to 4) were placed before the tags to increase sequence diversity and to ease base calling. We followed^56^ for PCR amplification conditions. Then, we performed purification and concentration using the MinElute PCR Purification Kit (Qiagen). Three PCR blanks were prepared by performing amplifications with the PCR mix in the absence of a DNA template. We prepared the libraries with the BIOO NEXTFLEX PCR-Free DNA-Seq Kit (Perkin-Elmer) and they were sequenced in a partial Illumina NovaSeq lane using 2 × 250 bp paired-end sequencing at Novogene Company.

### Metabarcoding pipeline and taxonomic assignment

For the bioinformatic analyses we followed the MJOLNIR2 pipeline scheme (https://github.com/uit-metabarcoding/MJOLNIR) that relies on the OBITools2^57^ software toolkit and other programs. Reads were paired with *illuminapairedend* and those with < 40 alignment quality score were discarded. Demultiplexing was performed with *ngsfilter* and the match of tags at each end was checked. We kept only reads between 310 and 319 bp and dereplicated sequences with *obiuniq*. Chimeras were detected and eliminated with the *Uchime de novo* algorithm from VSEARCH v1.4.1. Sequences were denoised with the DnoisE program^58^, which considers the variability in the three codon positions (https://github.com/adriantich/DnoisE). Alpha parameter was set to 4 and entropy values were obtained from the whole dataset to denoise each sample separately. With this procedure we generated Exact Sequence Variants (ESVs)^59^ that were clustered into molecular operational taxonomic units (MOTUs) with SWARM v3.0.0^60^ using d = 13^59^. Even if denoising is not strictly necessary for a MOTU-based approach as the one adopted here^59^, we nevertheless performed this step as ESVs are correct, reproducible sequences independent of the context of each study and are therefore the valid unit for comparisons across studies^59,61^. The representative sequences of MOTUs were taxonomically assigned with the May 2023 version of the COInr database (https://zenodo.org/records/7898363), which combines information from the BOLD and NCBI databases^62^. The COInr database is useful in metabarcoding studies of aquatic ecosystems, especially those targeting metazoan organisms. To avoid overclassification^63^, we eliminated from the database Insecta and Arachnida (but keeping Acariformes) using the select_taxa script^62^. Taxonomic assignment was performed using mkLTG from^64^. In short, mkLTG queries a database in BLAST format and seeks the lowest taxonomic group (LTG) to which a sequence can be assigned given a minimal similarity threshold and a set of parameters that should be met. Starting with high similarity values, if no LTG can be determined, the program tries the next threshold and associated parameter values. The parameter sets for each identity threshold used in this study are provided in Supplementary Table S1. After taxonomic assignment, we kept only MOTUs assigned to metazoans, and manually curated the database to eliminate non-marine organisms and contaminations.

Undetected errors still remain after the denoising procedures, making it necessary to perform further cleaning to refine the dataset. We applied the LULU procedure^65^ using a modification of the original function (https://github.com/jesuszarcero/LULU_corrected) to correct a bug previously detected (https://github.com/tobiasgf/lulu/issues/8). We then applied control and abundance filters following^54^. Finally, we deleted putative numts following^66,67^.

### Data analyses

All analyses were conducted in R version 4.0.2, mostly using the “vegan” package^68^. We computed rarefaction curves with *rarecurve* function and species abundance curves with *specaccum* function (Supplementary Figure S2). For each sample, we calculated the MOTU diversity (Shannon index) and evenness (Pielou index). A two-way ANOVA was used for both indices with the following fixed factors: *assemblage* (four levels: Not Restored, Restored Forest, Cala Rotja Forest and Miami Forest), *fraction* (two levels: Large and Small), and the interaction between them. The normal distribution of residuals was checked using Shapiro-Wilk tests and visually assessing quantile-quantile plots. The homogeneity of variances was checked using Levene’s tests and by means of the “residuals versus fitted” plot. We performed a 2D non-metric multidimensional scaling (nMDS) ordination analysis based on Bray-Curtis dissimilarities with the function *metaMDS* to visually represent the differences in MOTU composition among samples. We used as variable the relative read abundance of each MOTU in each sample (square-root transformed). Variability in sample MOTUs composition was measured with a multivariate PERMANOVA with the Bray-Curtis dissimilarity matrix (number of permutations = 999) using a two-factor design with *assemblage* and *fraction* as fixed factors, with the function *adonis2*. Pairwise tests were performed among levels of significant factors (https://github.com/pmartinezarbizu/pairwiseAdonis).

We also grouped the MOTUs into major metazoan phyla to compare the taxonomic assignment of our samples, and computed the relative abundance of reads and relative abundance of MOTUs per phylum of each assemblage and fraction. Moreover, we used the “mvabund” package^69^ to understand the drivers of invertebrate MOTUs composition. This package fits a generalised linear model (GLM) to each response variable (relative abundance of each MOTU in reads, in our case) with a set of predictor variables, which are then used to make community-level inferences using resampling-based hypothesis testing. To disentangle which variables had a stronger effect on the MOTUs composition, we included as predictor variables the biomass of the four most abundant macrophytes: *Cystoseira s.l.* species, *Cymodocea nodosa*, *Padina pavonica*, and *Halopteris scoparia*, categorised into five levels: 0, 1–25, 26–50, 51–75, and > 75 g of wet weight per quadrat (0.0625 m^2^). We used the negative binomial distribution and a log-link function with relative MOTU abundances as the variable. Model accuracy was assessed by a plot of residuals vs. fitted values. We performed the analyses considering the whole MOTUs dataset and for each of the most abundant metazoan phyla (i.e. Arthropoda, Mollusca, Annelida, Porifera, Chordata, and Cnidaria), to test whether the predictor variables had a different effect on the different invertebrate groups. Lastly, we also used the indicator value method (IndVal) from the “indicspecies” package^70^ to identify the MOTUs that were characteristic of the different assemblages. We set the Indval threshold to 0.4 and the p-value for significance at 0.02. The significance of indicator values was tested through random permutations (*n* = 999). For mvabund and IndVal analyses we used the MOTUs with relative abundances > 0.05% for each taxonomic group to focus on the most abundant MOTUs and to increase statistical power.

## Results

**Fig 1:**
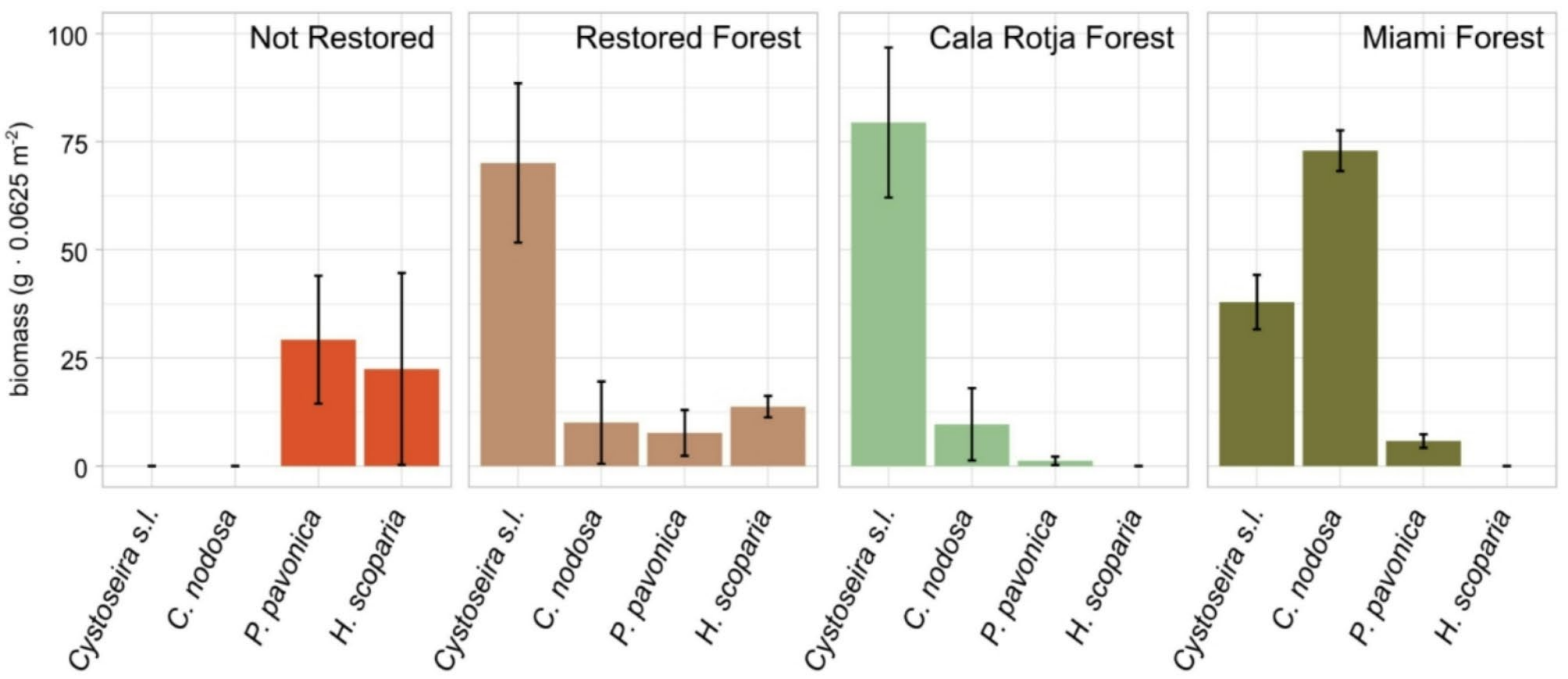
Biomass ± SD (wet weight per sample) of the most abundant macrophyte species found in each assemblage.

In the studied macroalgal assemblages, *Cystoseira s.l.* was the most abundant species, being present in Restored Forest, Cala Rotja Forest and Miami Forest, with 70 ± 21, 79 ± 19, and 38 ± 7 g per quadrat (0.0625 m^2^), respectively (mean ± SD, Fig. 1). Similarly, the seagrass *Cymodocea nodosa* was present in these three assemblages, but with higher abundance in Miami Forest (73 ± 5 g). These two species were absent in the Not Restored assemblage. The species *Padina pavonica*, on the contrary, was mainly found in the Not Restored assemblage (29 ± 17 g), together with *Halopteris scoparia* (22 ± 25 g). *H. scoparia* was also present in the Restored Forest (14 ± 3 g), but was absent in both Cala Rotja Forest and Miami Forest (Fig. 1). Likewise, *P. pavonica* was present at low abundances in the assemblages other than the Not Restored.

Considering all samples, we obtained a total of 106,034,539 reads from the sequencing run of the COI amplifications, and after the elimination of chimeras and singletons there were 89,560,138 reads remaining, corresponding to 1,890,086 ESVs after denoising. These ESVs were clustered by SWARM in 52,827 initial MOTUs. After taxonomic assignment, elimination of non metazoans and subsequent filters, the final COI dataset consisted of 943 MOTUs and 29,702,789 reads. The average number of reads per sample was 1,237,616 ± 1,952,792 (mean ± SD). Overall, 26.4% of MOTUs were taxonomically assigned to family or lower level, and they represented 41.5% of reads (30.6% of reads were identified at species level). The initial ESV table is presented in Supplementary File 1 in fasta format. The final MOTU table with taxonomic assignment, number of reads per sample, and representative sequences is provided in Supplementary File 2. All sequences generated in this study have been deposited in the Sequence Read Archive (SRA) under BIOPROJECT (PRJNA1179718).

**Fig 2:**
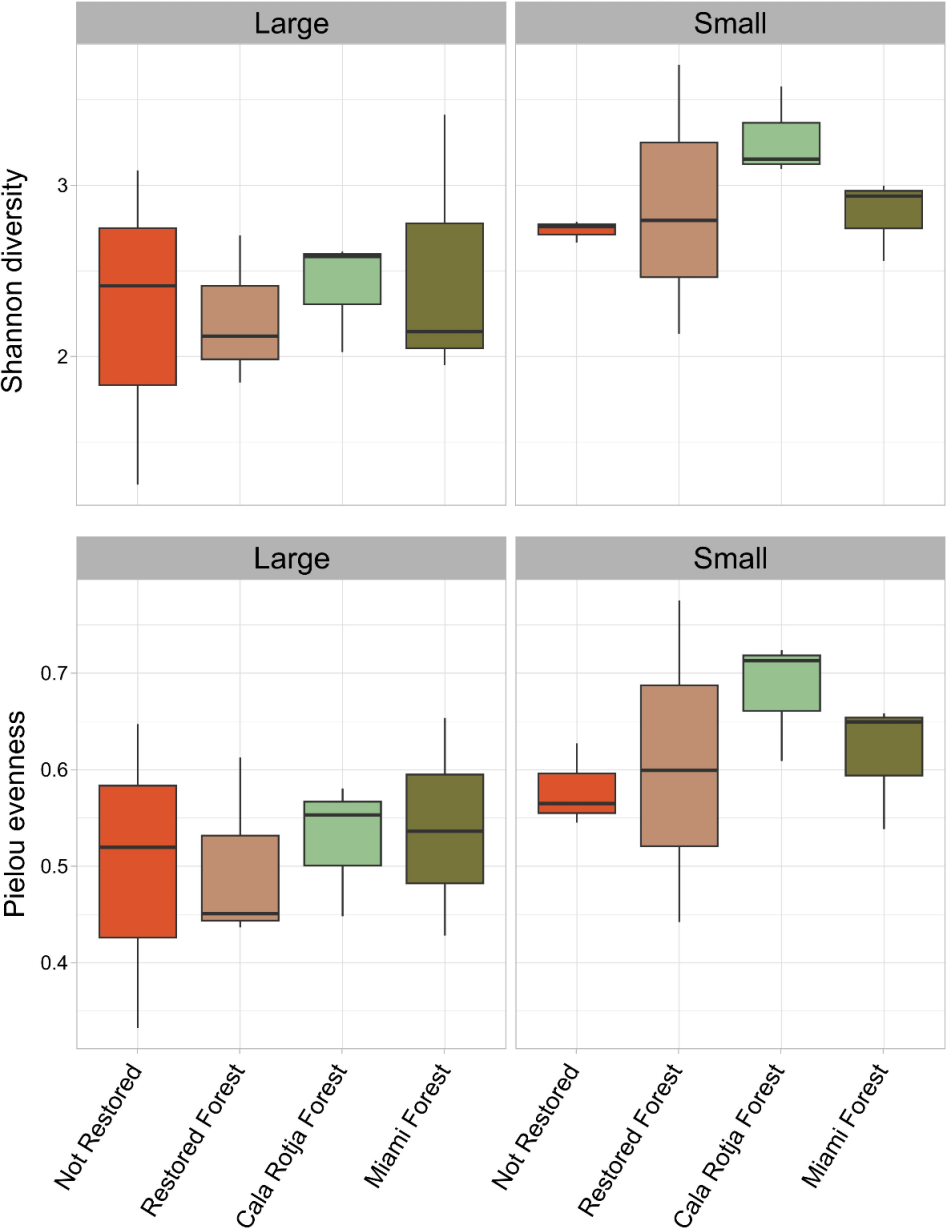
Macroinvertebrate MOTUs species diversity (measured with Shannon index) and species evenness (measured with Pielou index) among assemblages and fractions.

The two-way ANOVA indicated no significant differences in MOTU diversity (measured with the Shannon index) between assemblages, but it was significantly higher in the small fraction than in the large one (assemblage effect F_(3,16)_ = 0.443, *p* = 0.73; fraction effect F_(1,16)_ = 6.416, *p* = 0.022) (Fig. 2, Supplementary Table S2). Similarly, MOTU evenness (measured with Pielou’s index) did not differ between assemblages, but the distribution of MOTUs and their abundance was significantly more even in the small fraction than in the large one (assemblage effect F_(3,16)_ = 0.438, *p* = 0.73; fraction effect F_(1,16)_ = 5.741, *p* = 0.029) (Fig. 2, Supplementary Table S2). The interaction between both factors was not significant in any case (Supplementary Table S2).

**Fig 3:**
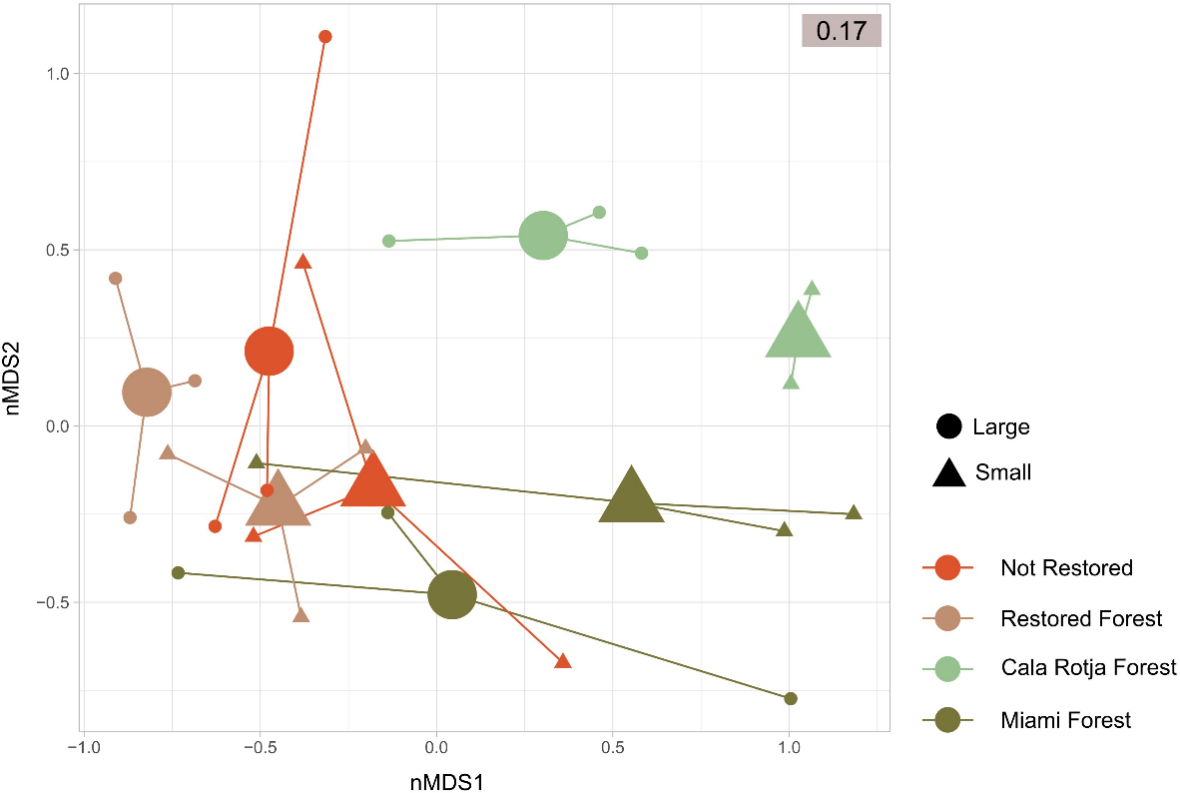
Non-metric multidimensional scaling (nMDS) plot displaying similarities among the macroinvertebrate MOTUs composition of the studied samples, using Bray-Curtis dissimilarity. MOTUs read abundance was fourth-root transformed prior to the analyses.

The nMDS ordination plot exhibited little separation among the centroids of the two fractions (i.e. Large vs. Small) that corresponded to the same assemblage (Fig. 3). In contrast, the diverse assemblages appeared at different positions over the first axis, albeit with noticeable overlap. The PERMANOVA results (Supplementary Table S3) showed differences in community composition between assemblages and between fractions (assemblage effect F_(3,23)_ = 2.640, *p* = 0.003; fraction effect F_(1,23)_ = 1.986, *p* = 0.038), while the interaction of these factors was not significant (*p* = 0.735, Supplementary Table S3). Pairwise tests of the assemblage factor showed that only Cala Rotja was significantly different from both the Restored Forest and the Not Restored assemblages.

**Fig 4:**
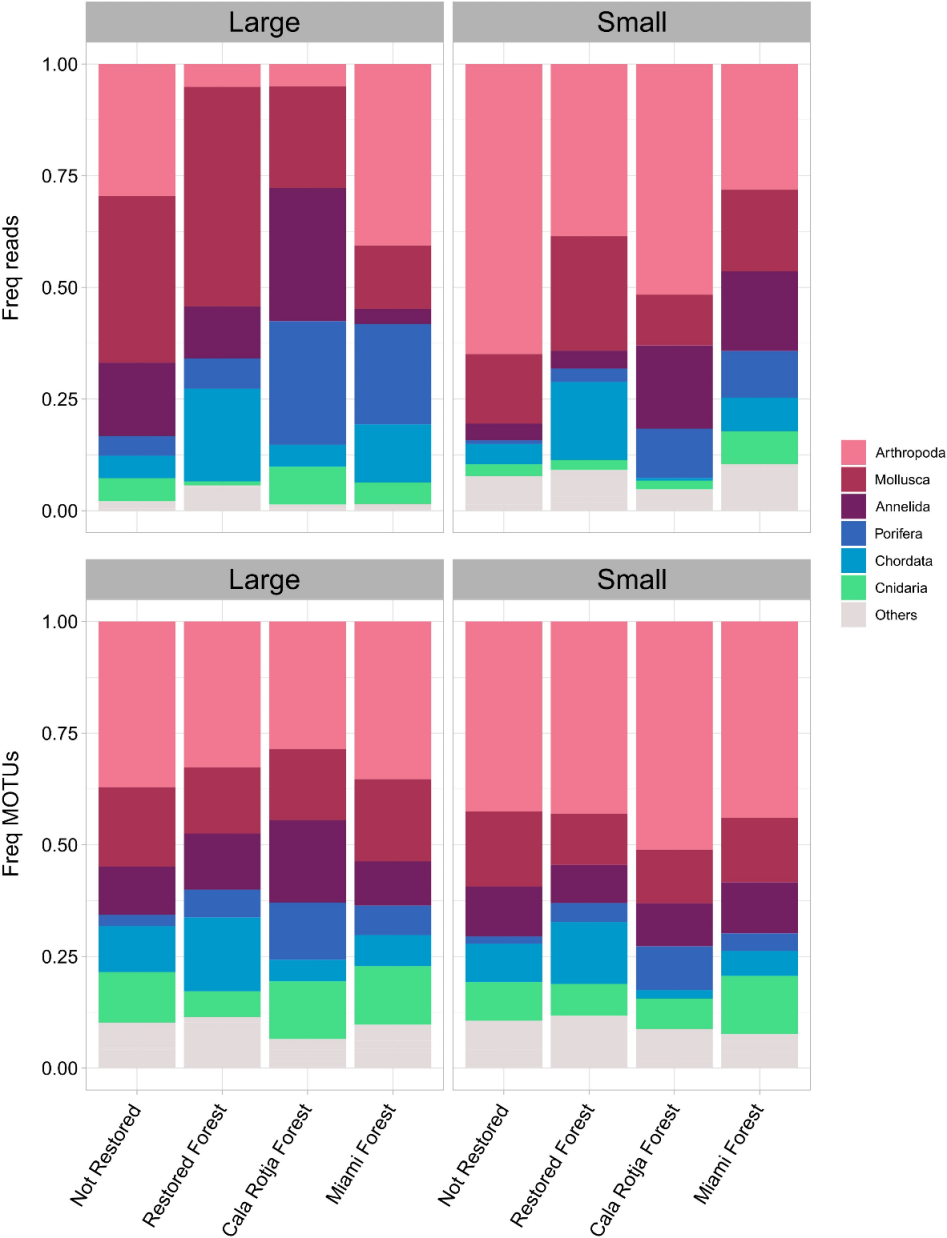
Frequency of read abundance and frequency of MOTUs per assemblage and fraction size belonging to the different phyla within Metazoa.

Taxonomic assignment of samples in terms of the relative number of reads and MOTUs of metazoans is presented in Fig. 4. The most MOTU rich and abundant phylum was Arthropoda (259 MOTUs, 32.7% of reads). In terms of MOTU richness it was followed by Mollusca (103 MOTUs, 28.9% of reads), Cnidaria (80 MOTUs, 3.7% of reads), and Annelida (71 MOTUs, 16.4% of reads). Arthropoda and Annelida were overall similarly present (based on the relative number of reads) in the four assemblages when considering both fractions (Fig. 4). Mollusca were especially abundant in Not Restored and Restored Forest, but they were also represented in Cala Rotja Forest and Miami Forest (Fig. 4). On the other hand, Porifera was more abundant in assemblages dominated by *Cystoseira s.l.* species, in particular in Cala Rotja Forest and Miami Forest (19.3 and 16.4% of reads, considering both fractions), but it was also present in the Restored Forest assemblage (4.9% of reads) and, with low abundance, in the Not Restored (2.6% of reads). Chordata (mostly ascidians) was more abundant in Restored Forest (19.1% of reads), followed by Miami Forest (10.3% of reads). Overall, we found more metazoan variability between assemblages in the relative frequency of reads rather than in the relative frequency of MOTUs (Fig. 4). In the Restored Forest, the most abundant MOTUs that could be assigned at the genus or species level with PID ≥ 95 were the molluscs *Bittium reticulatum*, *Chiton olivaceus* and *Jujubinus striatus*, the sponge *Dictyonella* sp. and the ascidian *Ascidia colleta*. In the Cala Rotja Forest, the most abundant MOTUs were the sponge *Biemna* sp. the echinoderm *Amphipholis squamata*, the annelid *Oxydromus pallidus*, the ascidian *Ecteinascidia turbinata*, and the cnidarian *Isozoanthus* sp. In the Miami Forest, the most abundant MOTUs were *Ecteinascidia turbinata*,* Biemna* sp., the sponge *Pione* sp., the pycnogonid *Endeis spinosa*, and *Jujubinus striatus*.

The results of the multivariate GLM approach showed that *Cystoseira s.l.* species, *Cymodocea nodosa* and *Halopteris scoparia* significantly influenced the overall MOTU composition, while *Padina pavonica* had no effect on it (Table 1). When performing the analysis for each main phylum, similar results were consistently obtained for Annelida, Porifera, Chordata, and Cnidaria (Table 1). However, the composition of Mollusca was influenced by *Cystoseira s.l.* species and *H. scoparia*, whereas Arthropoda composition was significantly explained by *H. scoparia* (Table 1).

**Table 1:** Outcome of the *mvabund* analysis of the effect of explanatory variables (biomass of the most abundant macrophyte species) on the MOTUs structure and composition. Results are presented for the whole MOTUs dataset and for each of the main phyla (i.e. Arthropoda, Mollusca, Annelida, Porifera, Chordata, and Cnidaria). The asterisk (*) indicates a significant p-value.

All MOTUs dataset	Res.Df	Df.diff	Dev	Pr(> Dev)
(Intercept)	23			
*Cystoseira s.l.* species	20	3	1,562.1	0.003*
*Cymodocea nodosa*	17	3	896.4	0.008*
*Padina pavonica*	16	1	190.4	0.282
*Halopteris scoparia*	14	2	682.2	0.001*

Arthropoda	Res.Df	Df.diff	Dev	Pr(> Dev)
(Intercept)	23			
*Cystoseira s.l.* species	20	3	473.2	0.066
*Cymodocea nodosa*	17	3	313	0.069
*Padina pavonica*	16	1	49.5	0.464
*Halopteris scoparia*	14	2	256.1	0.009*

Mollusca	Res.Df	Df.diff	Dev	Pr(> Dev)
(Intercept)	23			
*Cystoseira s.l.* species	20	3	222.24	0.037*
*Cymodocea nodosa*	17	3	154.09	0.086
*Padina pavonica*	16	1	59.82	0.053
*Halopteris scoparia*	14	2	133.4	0.023*

Annelida	Res.Df	Df.diff	Dev	Pr(> Dev)
(Intercept)	23			
*Cystoseira s.l.* species	20	3	262.52	0.004*
*Cymodocea nodosa*	17	3	151.88	0.024*
*Padina pavonica*	16	1	21.47	0.611
*Halopteris scoparia*	14	2	106.65	0.034*

Porifera	Res.Df	Df.diff	Dev	Pr(> Dev)
(Intercept)	23			
*Cystoseira s.l.* species	20	3	137.43	0.003*
*Cymodocea nodosa*	17	3	73.66	0.024*
*Padina pavonica*	16	1	9.12	0.249
*Halopteris scoparia*	14	2	104.71	0.001*

Chordata	Res.Df	Df.diff	Dev	Pr(> Dev)
(Intercept)	23			
*Cystoseira s.l.* species	20	3	163.5	0.011*
*Cymodocea nodosa*	17	3	129.5	0.022*
*Padina pavonica*	16	1	58.4	0.056
*Halopteris scoparia*	14	2	108.9	0.023*

Cnidaria	Res.Df	Df.diff	Dev	Pr(> Dev)
(Intercept)	23			
*Cystoseira s.l.* species	20	3	180.57	0.046*
*Cymodocea nodosa*	17	3	120.55	0.036*
*Padina pavonica*	16	1	38.49	0.268
*Halopteris scoparia*	14	2	109.89	0.022*

**Fig 5:**
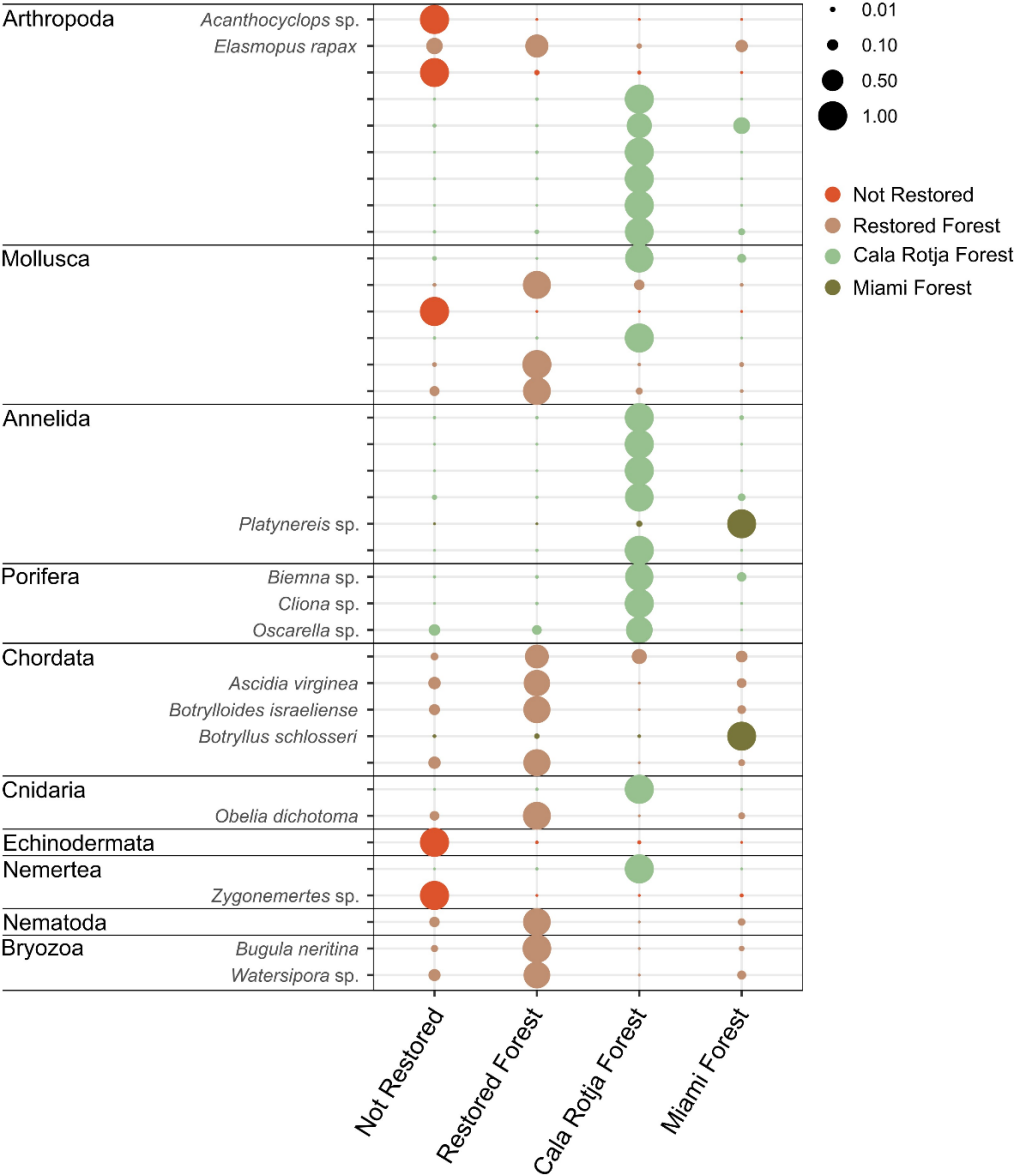
Characteristic MOTUs of the different assemblages obtained with the Indicator Value method (IndVal > 0.4; p-value < 0.02). The size of the circles represents the relative abundance of each MOTU within each assemblage type.

IndVal analyses revealed MOTUs significantly associated with the different assemblages (Fig. 5). The Restored Forest and Cala Rotja Forest assemblages had the highest number of indicator MOTUs (18 and 12 MOTUs, respectively), in comparison with the Not Restored and Miami Forest assemblages (5 and 2 MOTUs, respectively). Chordata and Bryozoa indicator MOTUs were mainly assigned to the Restored Forest, while Porifera and Annelida indicator MOTUs were mainly assigned to Cala Rotja Forest. Mollusca indicator MOTUs showed more variability in their assemblage assignment.

## Discussion

We present detailed information on the structure and composition of invertebrate communities obtained by metabarcoding of the COI gene, focusing on macroalgal assemblages in shallow and sheltered bays and representing two well-preserved forests, a restored forest, and a photophilous assemblage. Moreover, we explore how the identity and abundance of the most abundant macrophytes in each assemblage are related to changes in invertebrate communities, providing insights into macroalgae-invertebrate interactions.

Overall, the most abundant phyla obtained in the samples were Arthropoda, Mollusca and Annelida. These results are well aligned with classical identification methods based on taxonomy, which also report high abundance and diversity of these groups in macroalgal benthic assemblages (e.g^21,71–73^). Shannon diversity and Pielou’s evenness index computed with all metazoan MOTUs showed no differences among assemblages, but MOTUs composition was consistently more diverse and evenly distributed in the small fraction. This result is also in accordance with previous studies, which advise the size partitioning of the samples when dealing with highly complex and structured communities^52,74^. This step during sample processing ensures the representation of small sized groups and enhances their detection in the smaller fraction, often resulting in higher diversity. Shannon diversity averaged 2.6 ± 0.6 in our samples, similarly to values obtained with metabarcoding techniques in other Mediterranean macroalgal shallow-rocky communities in the Balearic Islands, with values of ca. 2.9^75^.

Regarding the relative abundance of MOTUs and reads of the different phyla within the different assemblages, we observed that groups such as Arthropoda and Annelida are on average equally present. However, Chordata (mainly dominated by ascidian species) was more abundant in the restored and Miami forested assemblages. In the same line, Porifera relative abundance was higher in both reference assemblages, followed by the restored one. These results show different patterns across assemblages depending on the specific taxa. If we consider together those groups with encrusting species or massive growth, such as Porifera and Chordata (mostly ascidians), it seems that their abundance increases in assemblages where *Cystoseira s.l.* species are present. Some sponges usually settle on the surface of *Cystoseira s.l.* species (e.g^76^), particularly in long-lived species and over the thallus, which is the part of the alga that persists over their annual cycle. Other studies have also found that *Cystoseira s.l.* canopies can provide suitable conditions for some sponge species, such as *Dysidea* sp.^77^, *Cliona* sp.^71^, *Crambe crambe*, *Sarcotragus spinulosa* and *Haliclona* sp^78^. All these species were found in this study only in samples belonging to the restored and reference assemblages, representing various taxonomic units (e.g. *Cliona* sp. accounted for 4 different MOTUs). Similarly, some ascidians were identified only in samples with *Cystoseira s.l.* species, such as *Ascidia colleta* and *Didemnum* sp. Species from the genus *Didemnum* have also been reported in presence of *Cystoseira s.l.* species^79^.

When considering the biomass of the macrophyte species of each sample, we found that the abundance of *Cystoseira s.l.* species, *Cymodocea nodosa* and *Halopteris scoparia* had an effect on the MOTU distribution. It is widely reported that invertebrate communities may differ depending on the identity of the structural species (e.g^80,81^). Studies dealing particularly with comparisons between Mediterranean *Cystoseira s.l.* species and other macroalgae found that, for example, the photophilous *H. scoparia* can also sustain considerable levels of molluscan abundance and diversity^82^, while the calcareous alga *Ellisolandia elongata* may harbour a diversity of crustaceans similar to other canopy forming species^83^. One potential reason behind this is the morphology and structural complexity of such algae. In the frame of our study, *Cystoseira s.l.* species and *H. scoparia* present a finely-branched form, in contrast with the other two main species. *C. nodosa* is characterised by grass-like leaves, while *P. pavonica*, presents semi-circular, fan-shaped fronds with concentric bands, offering a substantial surface area. The similar structural complexity provided by *Cystoseira s.l.* species and *H. scoparia* may have resulted in the high similarity of invertebrate communities in both Restored Forest and Not Restored assemblages (*H. scoparia* was present in them both). Mobile invertebrates with short life spans (< 2 years) such as amphipods, isopods and small gastropods may equally live within their canopy (e.g^82,84^), benefiting from the algal structure as potential refuge from predation or shelter from hydrodynamism.

Restoration efforts in the Bay of Maó had varying effects on the invertebrate community. Our results suggest that groups such as Arthropoda and Mollusca respond not only to the presence or absence of *Cystoseira s.l.* species, but also to other accompanying species. In fact, Arthropoda composition was only significantly influenced by *H. scoparia*, while Mollusca was significantly influenced by both *Cystoseira s.l.* species and *H. scoparia*. Other groups such as Porifera and Chordata appear to have benefited from the restoration of *Gongolaria barbata*, as their overall abundance is higher in the restored assemblage when compared to the non-restored assemblage. Other studies assessing the recovery of invertebrate communities following macroalgal restoration also reported changes in species composition, but in most of them the restored assemblages did not completely resemble reference ones. For example, the addition of a canopy-forming macroalga altered the species structure and composition of invertebrates 12 months after restoration, but due to the considerable annual variability detected, it was concluded that longer periods would likely be needed to fully understand the recovery process^35^. A second study reported that invertebrate richness and diversity did not fully compare to those of healthy and mature assemblages but, in this case, it was inferred that either an increase in predation by fishes was responsible for the decrease in invertebrates or that the potential sources for fauna dispersal were too far from the restoration site^34^. Similarly, differences in local factors may have driven the observed differences between restored and reference assemblages^33^, since it is known that invertebrate communities are influenced by several biotic and abiotic factors^85–87^. In this sense, factors varying locally among assemblages and bays such as type of substrate, sedimentation rates, or predation could have influenced the outcomes of this study, and further research considering a broader gradient in these variables is warranted.

The use of metabarcoding techniques is now widely applied in marine biodiversity assessments and monitoring. In light of our results, we underline several advantages and drawbacks of this technique as a tool for evaluating the success of marine forest restorations. For example, data outcomes allow for a joint analysis of taxa that are often studied separately in available literature, such as bryozoans and sponges (usually recorded with biomass or coverage) compared to polychaetes and gastropods (usually recorded in number of individuals) (e.g^21,88^). Moreover, metabarcoding enhanced the detection and identification of Hydrozoa species, such as *Obelia* sp. and *Eudendrium* sp., which may form small colonies over macroalgae. Similarly, small taxa belonging to meiofauna (metazoans between 45 and 500 μm, e.g. Nematoda, Nemertea, Copepoda) are often omitted in invertebrate assessments because of the challenges to properly sort, identify and quantify them. However, although meiofauna is an abundant and highly diverse component of benthic ecosystems, a large proportion of their species remains still unknown^89^ and often results in a considerable proportion of taxonomically unassigned MOTUs, thus highlighting a gap in the identification and genetic characterisation of some of these groups^90^.

On the other hand, factors inherent to the metabarcoding approach, such as the selection of genetic marker and primers, with their associated biases, may influence the taxonomic resolution obtained^91,92^. We used the COI gene, which has been shown to identify about 60–80% of the taxa present in a sample (e.g freshwater macroinvertebrates^93^; marine macroinvertebrates^94^) and a generalist primer pair shown in silico to efficiently amplify most metazoan groups^52^. Nevertheless, the remaining biases and the dependence upon reference databases for taxonomic assignment^40^, may have limited our ability to recover the full macroinvertebrate assemblages. That being said, significant barcoding efforts have been made in recent years to improve and populate databases with new species, making them progressively more comprehensive and updated^95^, so many of the MOTUs assigned here at high taxonomic levels can eventually get a more accurate identification in the future. We acknowledge that additional samples to identify the macroinvertebrate assemblages with traditional taxonomical methods would have provided a valuable comparison, helping us to understand the complete accuracy of our molecular outcomes. However, such a comparison was outside the scope of our study. Other works have compared metabarcoding and morphological results in the context of marine communities, and the common finding is that both methods represent different windows to the biodiversity present, with metabarcoding in general identifying more taxa, but that the general ecological patterns detected are coherent across methods^94,96,97^.

In our study, we recorded high variability among samples from the same assemblage (particularly in the Not Restored and Miami Forest), and the obtained accumulation curves did not plateau, suggesting that more replicates may be needed to capture the complete diversity of MOTUs. This requirement conflicts with the rationale of avoiding destructive methods when dealing with critical conservation status or threatened species in restoration frameworks, where less invasive techniques are preferred^98^. In this sense however, it was demonstrated that options such as the sampling of water column do not constitute a reliable alternative to assess the benthic structure of marine communities via metabarcoding^99^ and, therefore, their use in macroalgal restoration success evaluations is not advised. Nearly 75% of the obtained MOTUs (representing 58% of all reads) were identified to a taxonomic level higher than family. This lack of resolution in the taxonomic assignment hinders the possibility to obtain comprehensive information about functional aspects of the species found in the samples. Ecological analyses with a functional perspective often require a detailed identification of the species^100^, since commonly used traits in invertebrate functional assessments show high variability among classes, orders and even some families (e.g^101^).

Overall, our findings showcase the relevance of macroalgal species identity and their structural complexity in shaping their associated invertebrate communities. This shaping is dependent on the considered taxa, therefore suggesting that the restoration of *Gongolaria barbata* benefited sessile, massive-growing species such as sponges and ascidians, but had little influence on other groups such as crustaceans and mobile molluscs. These groups may as well easily colonise other algal species such as *H. scoparia*, which may perform a similar role in terms of habitat provisioning. Finally, the metabarcoding tool provided a general overview of the invertebrate assemblages in macroalgal habitats, efficiently identifying specific meiofauna taxa, but its use as an indicator for macroalgal restoration assessments needs further development and testing.

## Electronic supplementary material

Below is the link to the electronic supplementary material.

Supplementary Material 1

Supplementary Material 2

Supplementary Material 3

## Data Availability

The data and R code supporting the findings of this study are available in the Github digital repository (https://github.com/cgalobart/Macroalgal-restoration-metabarcoding). All sequences generated in this study have been deposited in the Sequence Read Archive (SRA) under BIOPROJECT (PRJNA1179718) and are available at the following link: https://www.ncbi.nlm.nih.gov/bioproject/PRJNA1179718.
