# Publisher Correction: Metabarcoding identifies macroalgal composition as a driver of benthic invertebrate assemblages in restored habitats

**DOI:** 10.1038/s41598-025-99223-1

**Published:** 2025-04-24

**Authors:** Cristina Galobart, Jesús Zarcero, Adrià Antich, Xavier Turon, Emma Cebrian

**Affiliations:** 1 Department of Marine Ecology, Centre d’Estudis Avançats de Blanes (CEAB-CSIC), Accés Cala Sant Francesc 14, Blanes, (17300) Spain; 2 NORCE Norwegian Research Centre AS and Bjerknes Centre for Climate Research, 22 Nygårdstangen, Bergen, (NO-5838) Norway

Correction to: *Scientific Reports* 10.1038/s41598-025-93327-4, published online 21 March 2025

The original version of this Article contained errors in Table 1. During production the data for species *Halopteris scoparia* was inadvertently omitted under the “All MOTUs dataset”. The correct and incorrect values appear below.

Incorrect:

**Table:** 

All MOTUs dataset	Res.Df	Df.diff	Dev	Pr (> Dev)
(Intercept)	23			
*Cystoseira s.l.* species	20	3	1,562.1	0.003*
*Cymodocea nodosa*	17	3	896.4	0.008*
*Padina pavonica*	16	1	190.4	0.282

Correct:

**Table:** 

All MOTUs dataset	Res.Df	Df.diff	Dev	Pr (> Dev)
(Intercept)	23			
*Cystoseira s.l.* species	20	3	1,562.1	0.003*
*Cymodocea nodosa*	17	3	896.4	0.008*
*Padina pavonica*	16	1	190.4	0.282
*Halopteris scoparia*	14	2	682.2	0.001*

The original Article has been corrected.

