# Supplementary material for: Metabarcoding identifies macroalgal composition as a driver of benthic invertebrate assemblages in restored habitats
Source: Sci Rep. 2025 Mar 21;15:9817. doi: 10.1038/s41598-025-93327-4 (PMC11928537; doi:10.1038/s41598-025-93327-4)
Supplement: Supplementary file 3 — Supplementary Material 3 [file 41598_2025_93327_MOESM3_ESM.pdf]

Supplementary Information

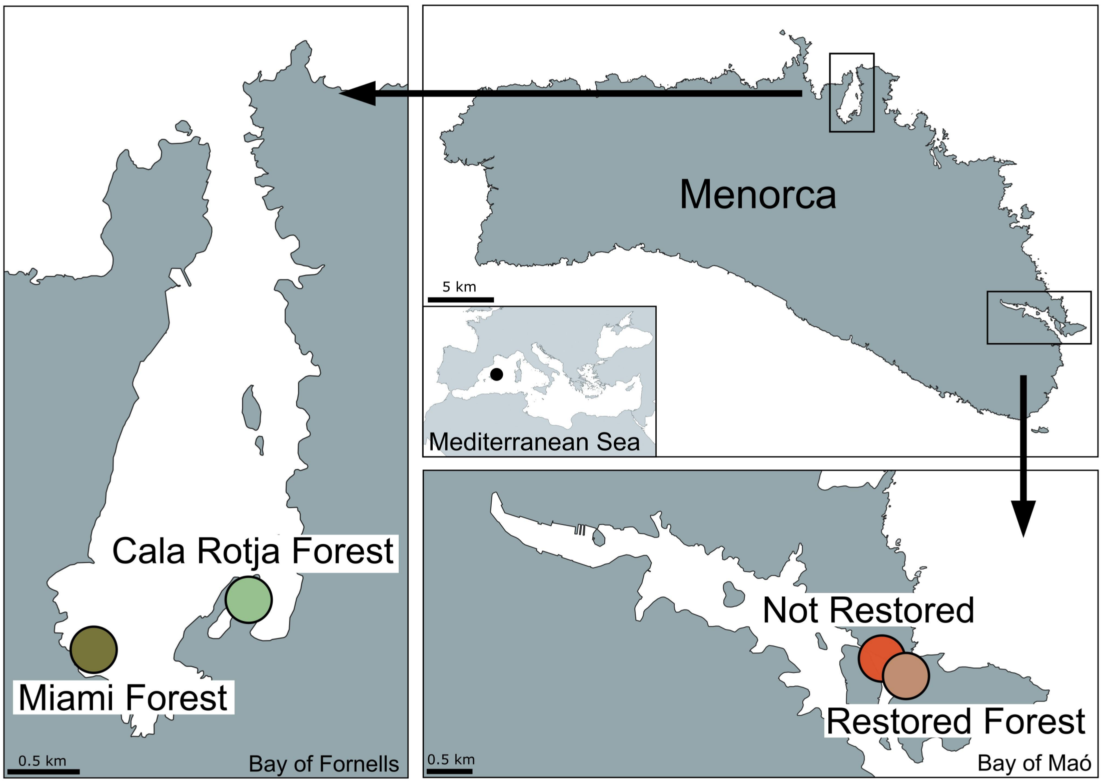

S Figure 1. Sampling sites for the four studied assemblages in Menorca Island, NW Mediterranean.

S Table 1. Parameter set used for the taxonomic assignment with mkLTG. See Meglécz et al. (2024) for the meaning of each parameter.

pid	pcov	phit	taxn	seqn	refres	ltgres
100	70	70	1	1	species	species
97	70	70	1	1	species	species
95	70	70	2	2	species	species
90	70	70	3	3	genus	species
85	70	70	4	4	family	genus
80	70	70	4	4	family	genus
75	70	70	5	5	order	family
70	70	70	5	5	phylum	order

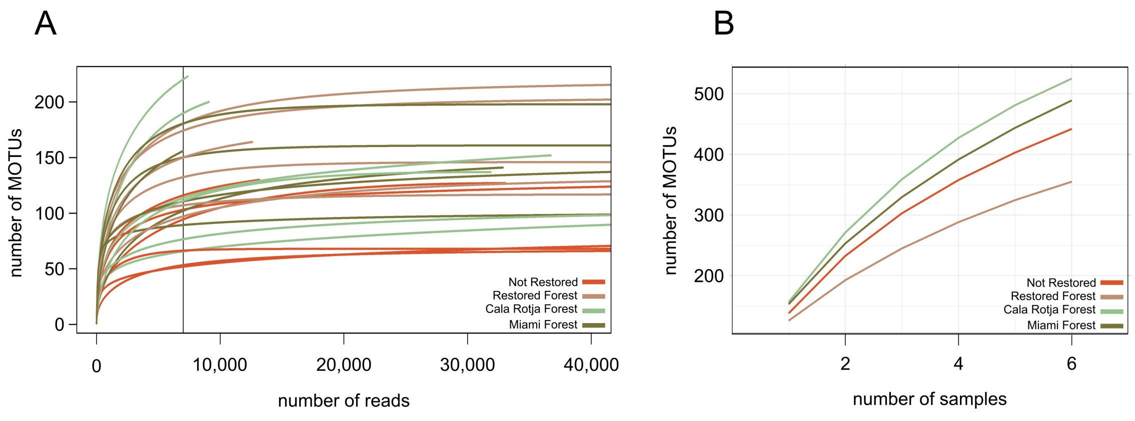

S Figure 2. (A) Rarefaction curves of the number of MOTUs at increasing numbers of reads for each sample (the sample with a smaller number of reads contained 7,013 reads), and (B) MOTU accumulation curves at increasing number of samples.

S Table 2. Two-way ANOVA summary of (A) MOTUs diversity measured with Shannon-Weiner index, and (B) MOTUs evenness measured with Pielou's index. The asterisk (*) indicates a significant p-value.

A) Shannon diversity

**ANOVA**	**Df**	**Sum Sq**	**R^2**	**F value**	**Pr (>F)**
assemblage	3	0.423	0.1411	0.443	0.73
fraction	1	2.045	2.0446	6.416	**0.0221***
assemblage:fraction	3	0.239	0.0798	0.250	0.86
residuals	16	5.098	0.3187		

B) Pielou's eveness

**ANOVA**	**Df**	**Sum Sq**	**R^2**	**F value**	**Pr (>F)**
assemblage	3	0.015	0.0049	0.438	0.73
fraction	1	0.065	0.0648	5.741	**0.0292***
assemblage:fraction	3	0.006	0.0020	0.175	0.91
residuals	16	0.181	0.0113		

S Table 3. (A) PERMANOVA results of the MOTUs composition with *assemblage*, *fraction*, and the interaction between them as fixed factors, using Bray-Curtis distances and 999 permutations. Relative MOTUs read abundance was fourth-root transformed prior to analyses. (B) Pairwise comparisons of *assemblage* factor. P-values of the post hoc test were adjusted with the Bonferroni method. The asterisk (*) indicates a significant p-value.

**PERMANOVA**	**Df**	**Sum Sq**	**R^2**	**F value**	**Pr (>F)**
assemblage	3	2.0019	0.2781	2.6405	**0.003***
fraction	1	0.5019	0.0697	1.9862	**0.038***
assemblage:fraction	3	0.6503	0.0904	0.8578	0.735
residual	16	4.0434	0.5618		
total	23	7.1976	1.0000		

**pair-wise *(assemblage)***	**Df**	**Sum Sq**	**F model**	**F value**	**p-value**	**p-adj**
Not Restored vs. Restored Forest	1	0.328	1.345	0.119	0.144	0.864
Not Restored vs. Cala Rotja Forest	1	0.872	3.382	0.253	0.004	**0.024***
Not Restored vs. Miami Forest	1	0.552	1.936	0.162	0.053	0.318
Restored Forest vs. Cala Rotja Forest	1	1.053	4.494	0.310	0.004	**0.024***
Restored Forest vs. Miami Forest	1	0.598	2.286	0.186	0.036	0.216
Cala Rotja Forest vs. Miami Forest	1	0.600	2.176	0.179	0.031	0.186
